# Error Analysis and Optimization of Structural Parameters of Spatial Coordinate Testing System Based on Position-Sensitive Detector

**DOI:** 10.3390/s24175740

**Published:** 2024-09-04

**Authors:** Haozhan Lu, Wenbo Chu, Bin Zhang, Donge Zhao

**Affiliations:** 1 College of Information and Communication Engineering, North University of China, Taiyuan 030000, China; s202205008@st.nuc.edu.cn (H.L.); zhangbinsmart@163.com (B.Z.); 2 College of Mechatronic Engineering, North University of China, Taiyuan 030000, China; icwb0707@163.com

**Keywords:** position-sensitive detector, remote measurement, spatial coordinate testing, structural parameters, precision analysis

## Abstract

For the research on real-time accurate testing technology for the explosion point spatial coordinate of munitions, its currently commonly used methods such as acoustic–electric detection or high-speed imaging are limited by the field conditions, response rate, cost, and other factors. In this paper, a method of spatial coordinate testing for the explosion point based on a 2D PSD (position-sensitive detector) intersection is proposed, which has the advantages of a faster response, better real-time performance, and a lower cost. Firstly, a mathematical model of the spatial coordinate testing system was constructed, and an error propagation model for structural parameters was developed. The influence of the position of the optical axes’ intersection as well as the azimuth angle and pitch angle on the test accuracy of the system was simulated and analyzed, thus obtaining the distribution and variation trend of the overall error propagation coefficient of the system. Finally, experiments were designed to obtain the test error of the system for validation. The results show that the system test accuracy is high when the azimuth angle is 20°–50°, the overall error propagation coefficient does not exceed 48.80, and the average test error is 56.17 mm. When the pitch angle is −2.5°–2.5°, the system has a higher test accuracy, with the overall error propagation coefficient not exceeding 44.82, and the average test error is 41.87 mm. The test accuracy of the system is higher when the position of the optical axes’ intersection is chosen to make sure that explosion points fall in the region of the negative half-axis of the $Z_{w}$-axis of the world coordinate system, with an overall error propagation coefficient of less than 44.78 and an average test error of 73.38 mm. It is shown that a reasonable selection of system structure parameters can significantly improve the system test accuracy and optimize the system deployment mode under the long-distance field conditions so as to improve the deployment efficiency.

## 1. Introduction

Spatial positioning technology has a wide range of applications in many fields, such as aerospace, military, agriculture, etc. [1,2,3,4], and especially occupies an important position in spacecraft attitude estimation [5,6] and munitions tracking and positioning [7,8,9], as well as robot navigation [10,11,12,13,14]. In recent years, the position testing of the explosion of munitions (hereinafter referred to as the explosion point) has been a hot spot of research, which is a crucial measurement parameter when evaluating munitions. High-precision coordinate testing for the explosion point provides important reference values for the development and effectiveness evaluation of equipment systems [15].

Currently, the most common methods of coordinate testing for the explosion point of munitions are the high-speed camera method [16,17,18] and the acoustic–optical test method [19,20,21]. The former is based on binocular stereo vision (BSV), where the explosion flare captured by two high-speed cameras is subjected to image processing and feature point extraction, and then 3D reconstruction is performed to obtain the explosion point coordinates. Wang proposed an image processing algorithm based on time and epipolar constraint based on BSV to measure and display the location of live ammunition explosion points relative to the bull’s eye [17]. The acousto-optic test method uses photodetectors or acoustic sensors to achieve the spatial positioning of explosion points. Cao proposed a measurement method of projectile explosion position acousto-optic compound detection using acoustic sensors and a compound-eye photoelectric detection sensor and verified the feasibility through theoretical analysis and live ammunition tests [20]. The position-sensitive detector (PSD) proposed in this paper is a non-contact photovoltaic device, which is based on the transverse photoelectric effect and obtains the position information of the measured object by solving the energy center of gravity of light. PSDs are currently used in a wide range of applications such as industrial inspection and aerospace and medical services [22,23]. Liu et al. worked on a graphene-based PSD motion tracking system, in which a light source is fixed on a target object and the target motion is continuously recorded by the PSD, thus realizing the highly sensitive real-time trajectory tracking of single or multiple targets [24,25,26]. Chien presented an integrated nanoelectromechanical in-plane displacement sensor based on a nanoelectromechanical trampoline resonator and sub-nanometer displacement detection realized by probing laser spots [27].

In the current research, the PSD is mainly applied in laboratory or outdoor close- range conditions, and the detection targets are mostly laser or LED light sources. In this paper, the PSD is applied to the field environment to realize the long-distance detection of explosion flares. Based on the principle of BSV, it is proposed to use two PSDs to intersect so as to realize the real-time positioning of the explosion point coordinates. In the test field, the explosion flare belongs to the dynamic target, and the high-speed camera needs to be controlled by an external trigger. External triggers, which are more expensive, require high sensitivity. The image information output by the camera is affected by the camera frame rate and pixel size and requires further image processing work, which is cumbersome. In addition, high-speed cameras are costly. Commonly used high-frame-rate cameras cost millions of dollars. For the acousto-optic test method, uncertain factors such as temperature, humidity, wind speed, and so on in the test field will affect the propagation quality of acoustic signals; obtaining effective acoustic signals from all kinds of interference sources is a key technology [19]. Moreover, conventional photoelectric detection requires a high photoelectric response rate and acquisition accuracy. Therefore, all of the above test techniques have certain shortcomings. Compared to high-speed cameras, which are array devices, PSDs have a continuous photosensitive surface with a position resolution of up to 1.4 μm for the spot. Compared to the pixel size of high-speed cameras, which is around 10 μm, PSDs have a higher position resolution. Currently, new PSDs combine 2D materials and semiconductors. The high mobility and absorbance of 2D materials provide PSDs with a high response speed and high sensitivity [28,29]. Therefore, PSDs are more accurate in capturing the moment of the explosion and have better real-time performance. PSDs directly output two-dimensional position information in the form of voltage value, which makes data processing more convenient. The sampling frequency of the system can be up to 2 MHz, which makes the data more abundant and complete. In addition, the randomness of the various factors that contribute to PSDs is relatively small in the test field. However, too strong background light will inevitably affect the detection accuracy of a PSD. It can be further processed to reduce the error caused by the impact of background light [30,31,32]. The PSD price of several companies in the market is currently USD 3000 to 5000, which is much lower in cost and easier to popularize.

In summary, the work carried out in this paper is as follows. According to the working principle of a 2D PSD and the principle of BSV, the mathematical model of a spatial coordinate testing system for the explosion point is first constructed. Then, the sources of error in the system model are analyzed and discussed, and the error propagation model of the influence of the optical axes’ intersection position, azimuth angle, and pitch angle on the system test accuracy is established. The distribution of the overall error propagation coefficient in the detection area of the system and the variation trend of the overall error propagation coefficient with respect to the azimuth angle and pitch angle are analyzed by simulation, and experimental verification is carried out. Finally, the optimal value range for the optical axes’ intersection position, azimuth angle, and pitch angle are given, which are of reference and guidance for the deployment of the system in long-distance field experiments. 

## 2. Principle of PSD-Based Spatial Coordinate Testing for the Explosion Point and Mathematical Model Establishment

**Figure 1:**
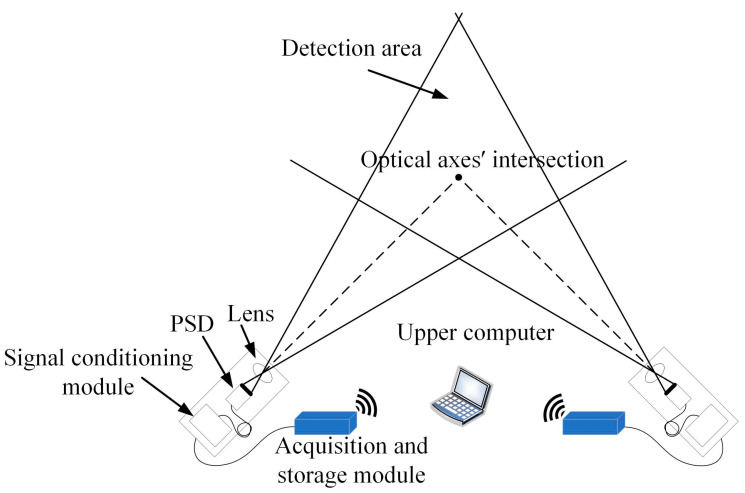
Composition of PSD-based spatial coordinate testing system for the explosion point.

The PSD-based spatial coordinate testing system for the explosion point is shown in Figure 1, which consists of the lens, a PSD, a signal conditioning module, an acquisition and storage module, and a data processing module on the upper computer.

A point near the actual explosion point is selected as the optical axes’ intersection of the two devices to form the detection area. The flare of the explosion point is converged by the optical lens to form a spot and then imaged on the photosensitive surface of the PSD. The position information is output through the signal conditioning module, then transmitted to the acquisition and storage module. The upper computer reads the collected signals through the wireless signal, which are finally substituted into the mathematical model of the PSD-based spatial coordinate testing system to obtain the spatial coordinate of the explosion point.

**Figure 2:**
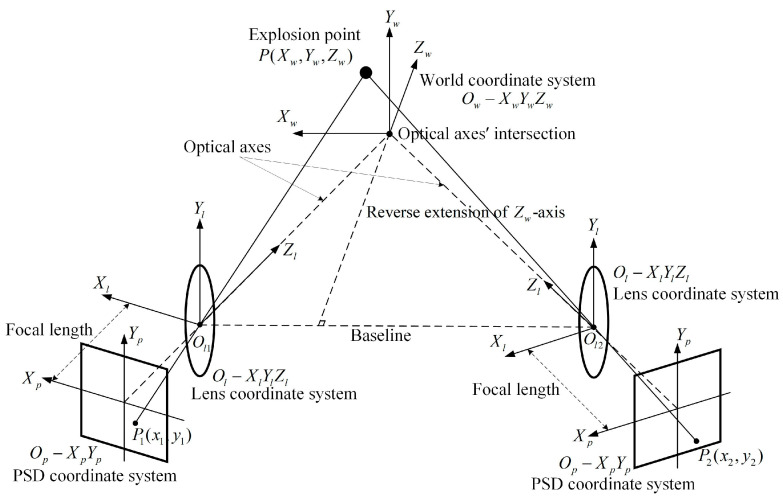
Principles of PSD-based spatial coordinate testing for the explosion point.

According to the basic principle of matrix transformation of the spatial coordinate system [33,34,35,36], the mathematical model of the PSD-based spatial coordinate testing system for the explosion point is constructed, as shown in Figure 2.

As shown in the figure, the world coordinate system $O_{w}-X_{w}Y_{w}Z_{w}$, the lens coordinate system $O_{l}-X_{l}Y_{l}Z_{l}$, and the PSD coordinate system $O_{p}-X_{p}Y_{p}$ are established separately. The origin of the PSD coordinate system is the center of the photosensitive surface, and the positive and negative semi-axes of the coordinate axis correspond to the positive and negative of the position information on the PSD. The lens coordinate system takes the center of the optical lens as the origin. The plane $X_{l}O_{l}Y_{l}$ is parallel to the PSD photosensitive surface, and the $Z_{l}$-axis is collinear with the PSD optical axis. The optical axes’ intersection is the origin of the world coordinate system. The $Y_{w}$-axis is perpendicular to the horizontal plane, and the plane $X_{w}O_{w}Z_{w}$ is parallel to the horizontal plane. $O_{l1}O_{l2}$ is the line connecting the centers of the two lenses, and the projection of $O_{l1}O_{l2}$ on the horizontal plane is defined as the baseline. The $X_{w}$-axis is parallel to the baseline. Let the point P be the actual explosion point, whose coordinate under the world coordinate system is $\left(X_{w},\;Y_{w},\;Z_{w}\right)$. Let points $P_{1}$ and $P_{2}$ be the spots imaged on the PSD photosensitive surface by the flare; Equation (1) can be calculated to obtain the coordinates of $P_{1}$ and $P_{2}$ as $\left(x_{1},\;y_{1}\right)$ and $\left(x_{2},\;{\;y}_{2}\right)$:

(1) $$\left\{\begin{array}{c} x=\frac{\left(V_{X2}+V_{Y1}\right)-(V_{X1}+V_{Y2})}{V_{X1}+V_{X2}+V_{Y1}+V_{Y2}}\times \frac{L}{2}\\ y=\frac{\left(V_{X2}+V_{Y2}\right)-(V_{X1}+V_{Y1})}{V_{X1}+V_{X2}+V_{Y1}+V_{Y2}}\times \frac{L}{2} \end{array}\right.$$

where $V_{X1}$, $V_{X2}$, $V_{Y1}$, and $V_{Y2}$ are the output voltage of the four electrodes of the PSD and L is the electrode length [37]. 

**Figure 3:**
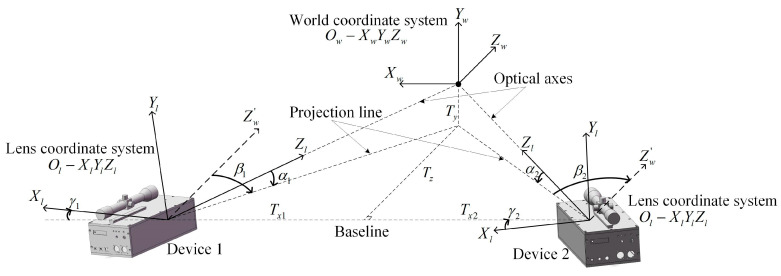
System structure parameters.

The explosion point coordinates are first converted from the world coordinate system to the lens coordinate system by performing a rigid motion, which changes only the spatial position (displacement) and orientation (rotation) of the object without changing its shape, as expressed in Equation (2):

(2) $$\left[\begin{array}{c} \begin{array}{c} X_{l}\\ Y_{l}\\ Z_{l} \end{array}\\ 1 \end{array}\right]=\left[\begin{array}{cc} R & -RT\\ 0 & 1 \end{array}\right]\left[\begin{array}{c} \begin{array}{c} X_{w}\\ Y_{w}\\ Z_{w} \end{array}\\ 1 \end{array}\right]$$

where $\left(X_{l},Y_{l},Z_{l}\right)$ represents the coordinate of the explosion point in the lens coordinate system. R is a rotation matrix which contains information about the angle of rotation of the lens coordinate system relative to the world coordinate system, and similarly, T represents the corresponding three-dimensional displacement vector, whose expressions are expressed as Equations (3) and (4):

(3) $$R=\left[\begin{array}{ccc} 1 & 0 & 0\\ 0 & cos\alpha & sin\alpha \\ 0 & -sin\alpha & cos\alpha \end{array}\right]\left[\begin{array}{ccc} cos\beta & 0 & sin\beta \\ 0 & 1 & 0\\ -sin\beta & 0 & cos\beta \end{array}\right]\left[\begin{array}{ccc} cos\gamma & sin\gamma & 0\\ -sin\gamma & cos\gamma & 0\\ 0 & 0 & 1 \end{array}\right]$$

(4) $$T=\left[\begin{array}{c} T_{x}\\ T_{y}\\ T_{z} \end{array}\right]$$

where α, β, and γ are the angles of rotation of the lens coordinate system $O_{l}-X_{l}Y_{l}Z_{l}$ relative to the world coordinate system $O_{w}-X_{w}Y_{w}Z_{w}$ around the *X*-axis, *Y*-axis, and *Z*-axis, which are defined as the pitch angle, azimuth angle, and roll angle, respectively. $T_{x}$, $T_{y}$, and $T_{z}$ are the corresponding displacement vector in the *X*-axis, *Y*-axis, and *Z*-axis directions, as shown in Figure 3.

where the dotted line $Z_{w}^{\prime }$-axis is parallel to the $Z_{w}$-axis. In the lens coordinate system $O_{l}-X_{l}Y_{l}Z_{l}$, looking from the positive direction of the $Y_{l}$-axis toward the origin, it is specified that the azimuth angle is positive when the coordinate system is rotated counterclockwise about the $Y_{l}$-axis. Looking from the positive direction of the $X_{l}$-axis toward the origin, the pitch angle is specified to be positive when the coordinate system is rotated counterclockwise about the $X_{l}$-axis.

Next is the conversion from the lens coordinate system to the PSD coordinate system, according to the similarity triangle calculation, which can be obtained as Equation (5), as follows:

(5) $$\rho \left[\begin{array}{c} x\\ y\\ 1 \end{array}\right]=\left[\begin{array}{cccc} f & 0 & 0 & 0\\ 0 & f & 0 & 0\\ 0 & 0 & -1 & 0 \end{array}\right]\left[\begin{array}{c} \begin{array}{c} X_{l}\\ Y_{l}\\ Z_{l} \end{array}\\ 1 \end{array}\right]$$

where ρ is the scale factor and f is the focal length of the lens; combining Equation (2) gives the following:

(6) $$\rho \left[\begin{array}{c} x\\ y\\ 1 \end{array}\right]=\left[\begin{array}{cccc} f & 0 & 0 & 0\\ 0 & f & 0 & 0\\ 0 & 0 & -1 & 0 \end{array}\right]\left[\begin{array}{cc} R & -RT\\ 0 & 1 \end{array}\right]\left[\begin{array}{c} \begin{array}{c} X_{w}\\ Y_{w}\\ Z_{w} \end{array}\\ 1 \end{array}\right]$$

According to Equations (1) and (6), the two devices provide two sets of mathematical relationships between the position information on the PSD and the coordinate of the explosion point in the world coordinate system. Through the least squares method, a set of optimal solutions can be calculated, thus acquiring the coordinate of the explosion point P in the world coordinate system.

## 3. Error Propagation Modeling for Structural Parameters and Deployment Optimization

When the devices are deployed in the blast effect field, the position of the optical axes’ intersection and the attitude of the device are both limited by the terrain conditions. How to efficiently deploy the device under the premise of guaranteeing the test accuracy of the explosion point coordinate is an important issue to be concerned about. Therefore, an error propagation model for the structural parameters of the system is developed in this paper, which focuses on analyzing the influence of the position of the optical axes’ intersection, azimuth angle, and pitch angle on the test accuracy of the explosion point coordinate, thus improving the system’s test accuracy and optimizing the deployment operation [38].

### 3.1. Error Source Analysis and Modeling

**Figure 4:**
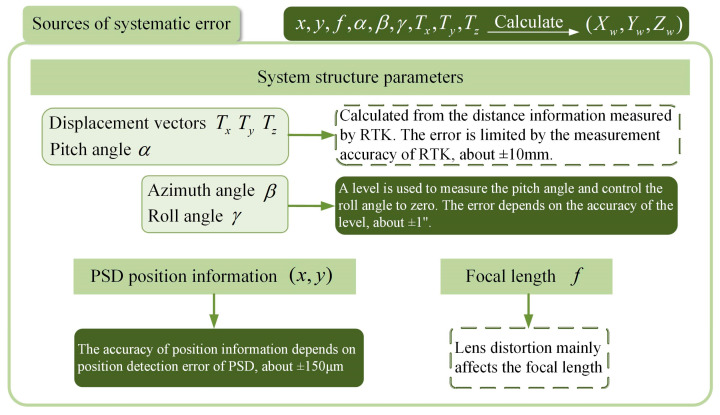
Analysis of sources of systematic error.

The expression for the coordinate of the explosion point P can be simply expressed as follows:

(7) $$P\left(X_{w},Y_{w},Z_{w}\right)=F(f_{i},x_{i},y_{i},\alpha_{i},\beta_{i},\gamma_{i},T_{xi},T_{yi},T_{zi})$$

where i = 1, 2 corresponds to device 1 and device 2, (x, y) is the spot coordinate on the PSD photosensitive surface, f is the focal length of the lens, α is the pitch angle, β is the azimuth angle, γ is the roll angle, and $T_{x},{\;T}_{y},\;T_{z}$ represent the displacement vectors. Based on these parameters, the sources of error in the system are analyzed as shown in Figure 4.

As can be seen from the figure, besides the error optimization of the structural parameters that are the focus of this paper, with respect to the PSD position detection error, due to the electrode structure and the photosensitive surface material of the PSD, there exists a nonlinear interval on the photosensitivity of the PSD, in which the position detection error of the PSD increases. The position detection error can be reduced by the nonlinear correction of the PSD [37]. Additionally, the distortion of the lens will affect the position of the imaging point, resulting in the effective focal length being different from the original focal length of the lens. The effective focal length can be determined by correcting for lens distortion [39,40], which consequently improves the positioning accuracy of the system.

By analyzing the sources of systematic error with respect to structural parameters, the roll angle can be adjusted to zero by using the precision level to simplify the mathematical model. The displacement along the positive direction of the coordinate axis is specified as positive, and $T_{z}$ = −100 m is set in order to analyze the test accuracy of the system at a distance of 100 m. The error propagation model of the system is shown in Equation (8):

(8) $$∆j=\sqrt{\sum_{k}{\left(\frac{\partial F_{j}}{\partial k}\cdot \delta_{k}\right)}^{2}}$$

where j = $X_{w},{\;Y}_{w},\;{\;Z}_{w}$, k represents the parameters $f,\;x,\;y,\;\alpha ,\;\beta ,\;\gamma ,{\;T}_{x},{\;T}_{y},\;T_{z}$ in Equation (7). $\frac{\partial F_{j}}{\partial k}$ is the error propagation coefficient for each parameter, which determines the variation trend of the error. $\delta_{k}$ is the absolute error value corresponding to each parameter, which affects the numerical magnitude of the error. In order to focus on the analysis of the distribution and variation trend of the error as well as to simplify the model, the absolute error value of each parameter is made equal uniformly, that is $\delta_{k}$ = δ = 0.01, so the following can be obtained:

(9) $$\frac{∆j}{\delta }=\sqrt{\sum_{k}{\left(\frac{\partial F_{j}}{\partial k}\right)}^{2}}$$

Let ∆ be the overall error of the system, which can be obtained by error synthesis:

(10) $$∆=\sqrt{{\left(∆X_{w}\right)}^{2}+{\left(∆Y_{w}\right)}^{2}+{\left(∆Z_{w}\right)}^{2}}$$

By combining Equations (9) and (10), the following can be obtained:

(11) $$\frac{∆}{\delta }=\sqrt{\sum_{k}{\left(\frac{\partial F_{X_{w}}}{\partial k}\right)}^{2}+\sum_{k}{\left(\frac{\partial F_{Y_{w}}}{\partial k}\right)}^{2}+\sum_{k}{\left(\frac{\partial F_{Z_{w}}}{\partial k}\right)}^{2}}$$

where ∆ is the overall error propagation coefficient of the system, which is proportional to the overall error. The overall error propagation coefficient is used instead of the overall test error to represent the error distribution and variation trend of the system. The distribution of the overall error propagation coefficient in the detection area and the variation trend of the overall error propagation coefficient with respect to the azimuth angle and pitch angle are derived from the simulation to analyze a reasonable range of values for the structural parameters.

### 3.2. Influence of Azimuth Angle on Test Accuracy and Optimization of Deployment

**Figure 5:**
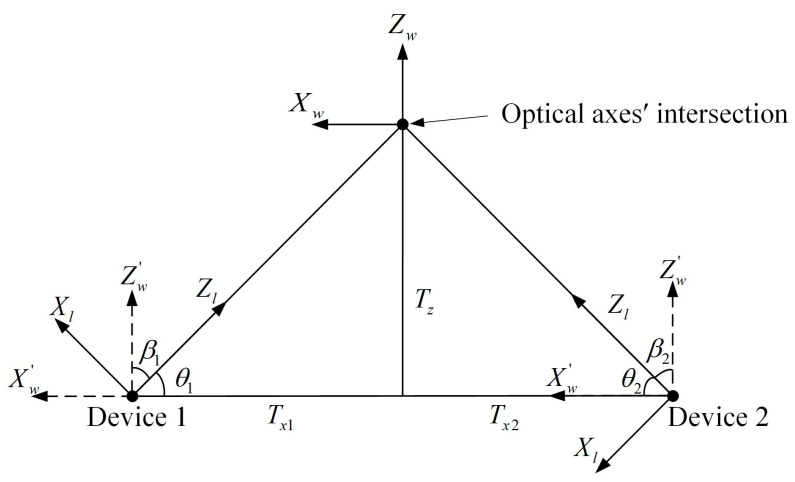
System deployment on the azimuth angle.

When the system is deployed in the field, the azimuth angle β plays an important role, which helps to determine the distance and direction of the device in advance. By simulating and analyzing the effect of the azimuth angle on the test accuracy of the system, the variation trend of the overall error propagation coefficient of the system with the azimuth angle can be obtained so that the degree of the azimuth angle with smaller errors can be selected as a way to improve the test accuracy of the system. A schematic of the deployment with respect to the azimuth angle is shown in Figure 5.

In the figure, $\beta_{1}$ and $\beta_{2}$ are the azimuth angles of the two devices. $\theta_{1}$ and $\theta_{2}$ are the angles between the optical axes and the baseline and are complementary to the corresponding azimuth angle. Then, $T_{x1}$ and $T_{x2}$ can be obtained:

(12) $$\left\{\begin{array}{c} T_{x1}=-T_{z}cot(\frac{\pi }{2}-\beta_{1})\\ T_{x2}=T_{z}cot(\frac{\pi }{2}+\beta_{2}) \end{array}\right.$$

In order to independently analyze the effect of the azimuth angle on the test accuracy of the system, it is necessary to control other variables. Then, the pitch angle is set to α = 0, the displacement is set to $T_{y}$ = 0, and the coordinate of the explosion point is set to (0, 0, 0), and substituting the parameters into Equation (6) gives the following:

(13) $$\left\{\begin{array}{c} x_{1}=-\frac{sin\beta_{1}+cos\beta_{1}cot(\beta_{1}-\frac{\pi }{2})}{20[cos\beta_{1}-sin\beta_{1}\cot\left(\beta_{1}-\frac{\pi }{2}\right)]}\\ x_{2}=-\frac{sin\beta_{2}+cos\beta_{2}cot(\beta_{2}+\frac{\pi }{2})}{20[cos\beta_{2}-sin\beta_{2}\cot\left(\beta_{2}+\frac{\pi }{2}\right)]}\\ y_{1}=0\\ y_{2}=0 \end{array}\right.$$

**Figure 6:**
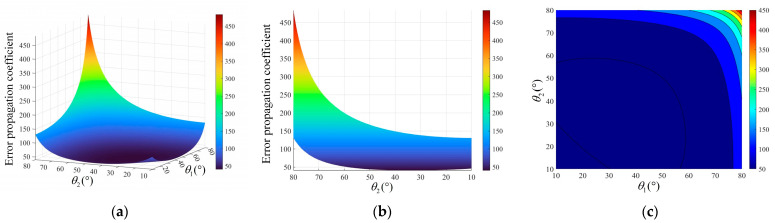
Error propagation coefficient with respect to the azimuth angle. (**a**) Overall distribution; (**b**) side view; (**c**) top view.

In order to more intuitively represent the effect of the azimuth angle on the error, θ is used here instead of the azimuth angle β as the independent variable for the analysis. Then, by substituting the above parameters and expressions into Equation (11), we can obtain the distribution and variation trend of the overall error propagation coefficient about $\theta_{1}$ and $\theta_{2}$ in the range of 10°–80°, as shown in Figure 6.

From the figure, it can be seen that the overall error propagation coefficient has a significant variation from 51.46 to 483.75 when $\theta_{1}$ and $\theta_{2}$ are between 50° and 80°, and the coefficient is maximal when $\theta_{1}$ = $\theta_{2}$ = 80°. As $\theta_{1}$ and $\theta_{2}$ get smaller, the overall error propagation coefficient decreases gradually. When $\theta_{1}$ and $\theta_{2}$ are taken from 20° to 50°, the overall error propagation coefficient is in the range of 40.63 to 48.80, which is small and does not vary significantly. The overall error propagation coefficient is minimal at $\theta_{1}$ = $\theta_{2}$ = 34.7°. Thereafter, the error propagation coefficient shows a certain increase and increases to 85.30 when $\theta_{1}$ = $\theta_{2}$ = 10°. Therefore, when $\theta_{1}$ and $\theta_{2}$ are taken from 20° to 50°, the overall error propagation coefficient is smaller and does not exceed 48.80, which corresponds to a higher test accuracy of the system.

### 3.3. Influence of Pitch Angle on Test Accuracy and Optimization of Deployment

It is ideal that the optical axes of both devices intersect horizontally at the time of deployment, but zeroing the pitch angle and roll angle at the same time is difficult to achieve because of the variable terrain conditions in the field. To zero angles at the same time, both devices need to be at the same altitude, which will greatly reduce the efficiency of the deployment, so it is preferable for the pitch angle to be taken into consideration in the mathematical model. However, the increase in the parameter will inevitably affect the test accuracy of the system. Therefore, it is necessary to analyze the effect of the pitch angle on the test accuracy of the system to obtain the optimal pitch angle range.

**Figure 7:**
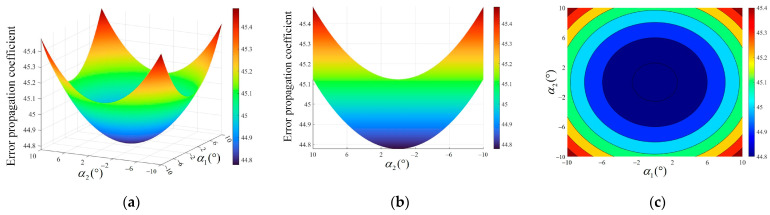
Error propagation coefficient with respect to the pitch angle. (**a**) Overall distribution; (**b**) side view; (**c**) top view.

Let the coordinate of the explosion point be (0,0,0), and set $\beta_{1}$ = 45°, $\beta_{2}$ = −45°, $T_{x1}$ = 100 m, and $T_{x2}$ = −100 m. The geometric relationship gives $T_{y}$ = 100$\sqrt{2}sin\alpha$. Then, Equation (14) is obtained by Equation (6):

(14) $$\left\{\begin{array}{c} x_{1}=0\\ x_{2}=0\\ y_{1}=-\frac{sin\alpha_{1}-cos\alpha_{1}tan\alpha_{1}}{20(cos\alpha_{1}+sin\alpha_{1}tan\alpha_{1})}\\ y_{2}=-\frac{sin\alpha_{2}-cos\alpha_{2}tan\alpha_{2}}{20(cos\alpha_{2}+sin\alpha_{2}tan\alpha_{2})} \end{array}\right.$$

where $\alpha_{1}$ is the pitch angle of device 1 and $\alpha_{2}$ is the pitch angle of device 2. Substituting the parameters and Equation (14) into Equation (11), the distribution and variation trend of the overall error propagation coefficient of the system about the pitch angle $\alpha_{1}$ and $\alpha_{2}$ in the range of −10° to 10° can be obtained, as shown in Figure 7.

As can be seen in Figure 7, the overall error propagation coefficient is symmetrically distributed about 0° when the pitch angle is between −10° and 10°, and the variation is not significant. The overall error propagation coefficient is taken to a minimum value of 44.78 for both sides of the device at a pitch angle of 0°. The overall error propagation coefficient increases gradually with the increase in the pitch angle, where the maximum is 45.48. Therefore, the pitch angles $\alpha_{1}$ and $\alpha_{2}$ are selected to be from −2.5° to 2.5°, at which time the overall error propagation coefficient is small and does not be more than 44.82, and the test accuracy of the system is higher.

### 3.4. Error Distribution in the Detection Area and Optimization of Deployment

The optical axes’ intersection in the system model is generally in the vicinity of the predicted explosion point, and the system forms the detection area with the optical axes’ intersection point as the center. By simulating the error distribution in the detection area, the area with the highest test accuracy can be found, and the coordinate testing of the system will be more accurate when the explosion points fall in this area. The test accuracy of the system is improved by determining a more optimal position of the optical axes’ intersection.

The pitch angle α and displacement $T_{y}$ are set to 0, and $\beta_{1}$ = 45°, $\beta_{1}$ = −45°, $T_{x1}$ = 100 m, and $T_{x2}$ = −100 m are set. By substituting the set parameters into Equation (6), Equation (15) can be obtained:

(15) $$\left\{\begin{array}{c} x_{1}=-\frac{X_{w}+Z_{w}}{20\left(Z_{w}-X_{w}\right)+4000}\\ x_{1}=-\frac{X_{w}-Z_{w}}{20\left(Z_{w}+X_{w}\right)+4000}\\ y_{1}=0\\ y_{2}=0 \end{array}\right.$$

**Figure 8:**
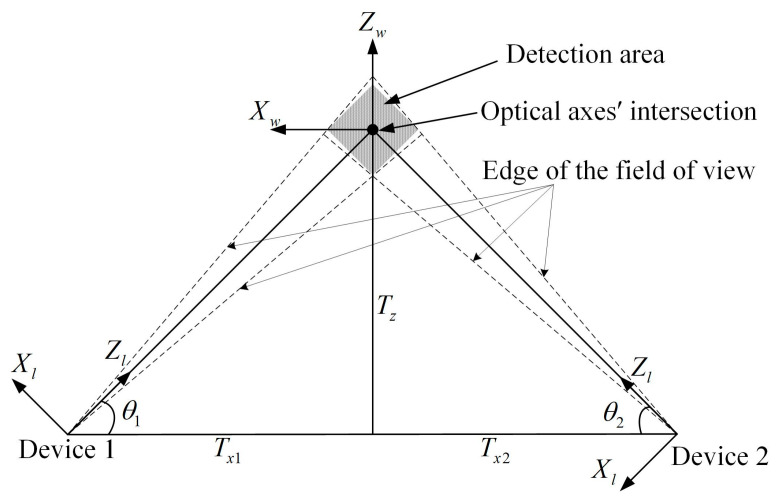
Detection area of the system.

Based on the pinhole imaging model, it can be calculated that the detection area formed by the system at a distance of $T_{z}$ = −100 m is approximately 800 m^2^, as shown in the shaded area in Figure 8.

**Figure 9:**
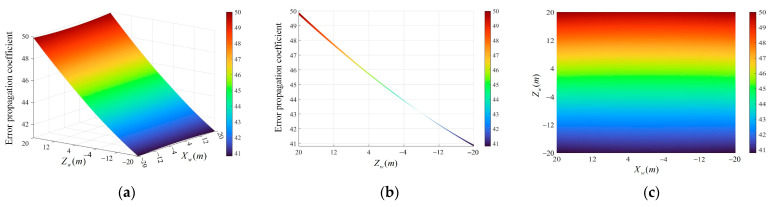
Error propagation coefficient in the detection area. (**a**) Overall distribution; (**b**) side view; (**c**) top view.

According to the relationship between the detection area and the constructed world coordinate system, analyzing the distribution and variation trend of the error propagation coefficient of $X_{w}$ and $Z_{w}$ in the range of −20 m to 20 m, Figure 9 is obtained through Equation (11).

**Figure 10:**
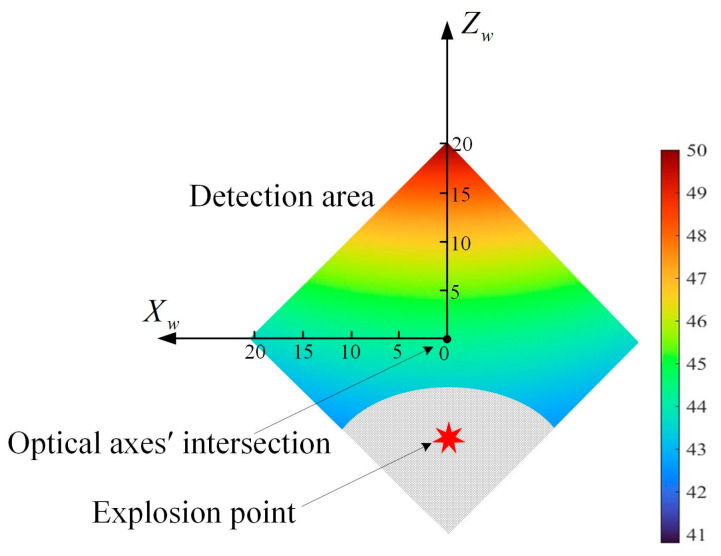
Relationship between the position of the explosion point and the optical axes’ intersection.

From Figure 9, the overall error propagation coefficient decreases gradually from 49.92 to 40.80 along the $Z_{w}$-axis of the world coordinate system from positive to negative. The amplitude of the change in the overall error propagation coefficient along the $X_{w}$-axis direction is largely insignificant, with a maximum difference of only 0.18. In the region of the negative half-axis of the $Z_{w}$-axis, the overall error propagation coefficient is small, with an overall error propagation coefficient of less than 44.78. Combining the top view with the detection area of the system gives Figure 10.

As shown in the figure, in the detection area, when the explosion points fall in the region of the negative half-axis of the $Z_{w}$-axis, which is the spot region in the figure, the overall error propagation coefficient is smaller, and the test accuracy of the system is higher. Moreover, the closer to the edge of the detection area, the smaller the error propagation coefficient, and it is taken to a minimum of 40.91 at the point $\left(X_{w},Z_{w}\right)$ = (0, −20). The selection of the position of the optical axes’ intersection should take into account the size of the detection area, the terrain conditions, and the size of the flare of the explosion point. It is important to avoid the flare of the explosion point extending beyond the detection area, thus affecting the accuracy of the spot position on the PSD. Choosing a suitable position of the optical axes’ intersection to make sure that explosion points fall in the region of the negative half-axis of the $Z_{w}$-axis of the world coordinate system can not only guarantee that the flare can be completely imaged on the photosensitive surface but can also realize the high precision of the system to test the spatial coordinate of the explosion point.

## 4. Experimental Protocol Design and Validation Analysis

### 4.1. General Experimental Protocol

**Figure 11:**
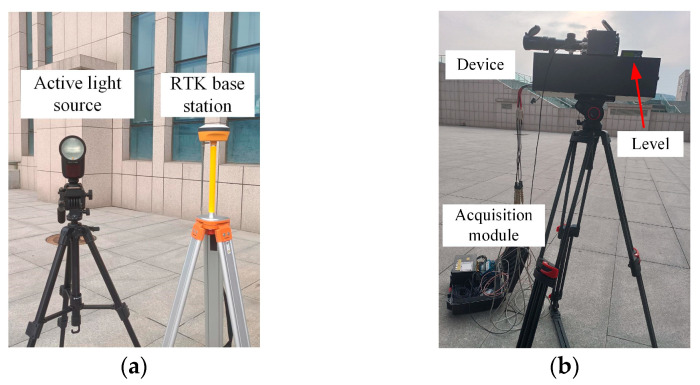
Components of the test system. (**a**) Active light source and RTK base station; (**b**) device and acquisition module.

In response to the simulation results of the error propagation model, validation experiments are designed regarding the test error distribution in the detection area and the effect of the azimuth angle and pitch angle on the test error. The system used for the test consists mainly of the device, acquisition module, measuring instrument with RTK technology, and an active light source for simulating the flare of the explosion point. The system components are shown in Figure 11. And the main instruments and devices used in the system are shown in Table 1.

**Figure 12:**
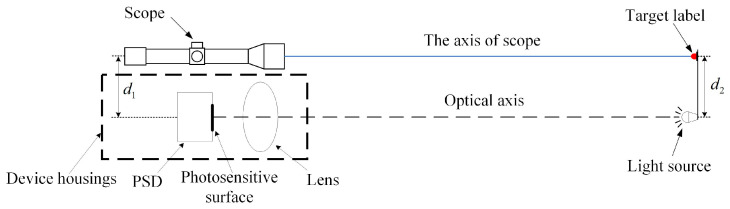
Principle of intersection assisted with a scope.

For the construction of the model and the calibration of the parameters, a method of using a scope to assist the optical axis to accomplish the intersection is proposed, as shown in Figure 12.

As shown in the figure, under laboratory conditions, the coordinates of the light source on the photosensitive surface detected by the PSD are used to adjust the scope to aim at the target so that $d_{1}$ = $d_{2}$, thus calibrating the axis of the scope to be parallel to the optical axis of the device. During deployment, optical axes may also intersect at one point by aiming at the same point with the scope. Then, system parameters such as the distance and angle are measured in combination with RTK instruments to realize rapid calibration under field conditions.

This is limited by factors such as the distance at which the light source can elicit a response from the device, the angular range at which the light source radiates, and the height range at which the device can be adjusted. The test distance designed for the experiment is smaller than that in the simulation model. The overall magnitude of the error obtained from the experiment will be affected as a result, but the distribution and variation trend of the error will remain consistent.

### 4.2. Validation Experiment for the Effect of Azimuth Angle on Test Accuracy

**Figure 13:**
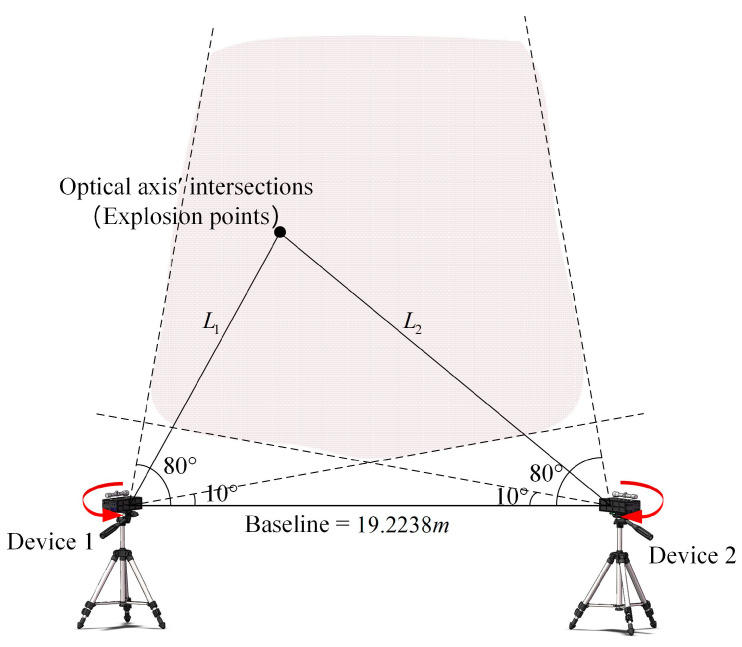
Schematic diagram of the experiment on azimuth angle. The arrows mean that the device was rotated horizontally during the experiment.

Based on the parameters in the error propagation model, the deployment of the validation experiment is shown schematically in Figure 13.

In the figure, the red area shows the intersection range of $\theta_{1}$ and $\theta_{2}$ from 10° to 80°. The light source used to simulate the explosion point is taken as the position of the optical axes’ intersection, thus controlling that the coordinate of the explosion point $\left(X_{w},{\;Y}_{w},{\;Z}_{w}\right)$ is always (0, 0, 0). The experiment procedure is as follows:

(1) Erect two devices, use a level to control the pitch angle and roll angle of the devices to zero, and use the RTK to measure the length of the baseline, then hold the position constant;
(2) Place a light source anywhere in the red area, aim both devices at the center of the light source to achieve optical axes’ intersection, and measure the length of $L_{1}$ and $L_{2}$, then calculate the azimuth angle;
(3 Use the flash trigger to trigger the light source, use the PSD to detect the position information of the flash, and download the waveform file to the host computer through the acquisition module;
(4) Repeat steps 2 and 3 to achieve a change in the azimuth angle of the system by continuously changing the position of the optical axis intersection point in the red region;
(5) Substitute the corresponding parameters of each group and the position information from the PSD into the mathematical model, then calculate the coordinate of the explosion point $\left(X_{c},{\;Y}_{c},{\;Z}_{c}\right)$.

The system test error E for each group is calculated by Equation (16):

(16) $$E=\sqrt{{\left(X_{c}-X_{w}\right)}^{2}+{{(Y}_{c}-Y_{w})}^{2}+{\left(Z_{c}-Z_{w}\right)}^{2}}$$

By replacing the azimuth angles $\beta_{1}$ and $\beta_{2}$ with the angles $\theta_{1}$ and $\theta_{2}$ as independent variables, the corresponding test error of the system for different angles can be obtained, as shown in Table 2.

**Figure 14:**
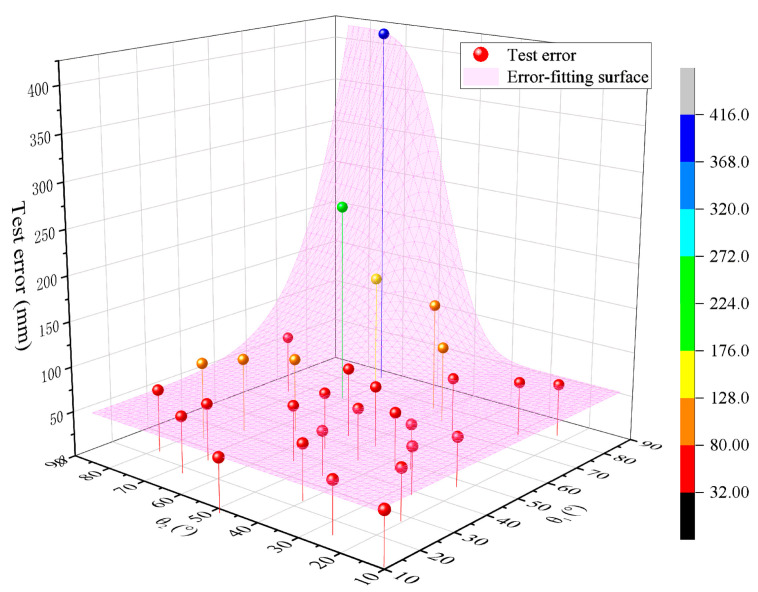
Distribution of test error on azimuth angle.

It can be obtained from the table that, at the condition of the baseline being 20 m, the maximum test error of the system is 415.12 mm, corresponding to 85.9585° and 72.8724° for $\theta_{1}$ and $\theta_{2}$, respectively. When $\theta_{1}$ is 34.316° and $\theta_{2}$ is 45.4239°, the system has the lowest test error of 50.17 mm. Combining the data in the table gives the distribution of the test error, as shown in Figure 14.

In the figure, the bubble points are the test error of the system when $\theta_{1}$ and $\theta_{2}$ are taken at different angles, and the pink surface fits the bubble points. The test error decreases gradually as $\theta_{1}$ and $\theta_{2}$ become smaller, and when $\theta_{1}$ and $\theta_{2}$ are larger than 50°, the change amplitude of the test error is large, while the variation in the test error is small for less than 50°. Combined with Figure 6 derived from simulation, it can be verified that the system test error is basically consistent with the variation trend of the error propagation coefficient obtained from simulation. The system test error is smaller when $\theta_{1}$ and $\theta_{2}$ are in the range of 20°–50°, and the system test accuracy is higher with an average test error of 56.17 mm.

### 4.3. Validation Experiment for the Effect of Pitch Angle on Test Accuracy

**Figure 15:**
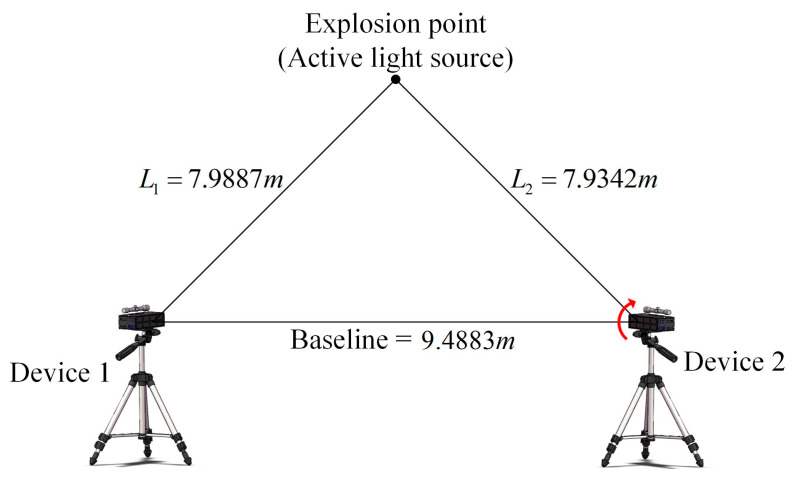
Schematic diagram of the experiment on pitch angle. The arrow means that the device was rotated vertically during the experiment.

Regarding the verification of the effect of the pitch angle on the test accuracy of the system, the test schematic is shown in Figure 15.

According to the schematic, the light source used to simulate the explosion point is also taken as the position of the optical axes’ intersection, to control the coordinate of the explosion point $\left(X_{w},Y_{w},Z_{w}\right)$ to be (0, 0, 0). The specific experiment steps are as follows:

(1) Set up two devices and light sources, use a level to control the roll angle of the devices to zero, use RTK to measure the length of $L_{1}$ and $L_{2}$ and baseline, then calculate the azimuth angle, keep the position constant to control the azimuth angle constant;
(2) Adjust the height of the device 1 on the left side to aim at the center of the light source at a certain angle, record the pitch angle of the device 1 with a level, and keep the attitude unchanged;
(3) Arbitrarily change the height of device 2 within the adjustable range and aim at the center of the light source, then record the pitch angle with a level;
(4) Use the flash trigger to trigger the light source, use the PSD to detect the position information of the flash, and download the waveform file to the host computer through the acquisition module;
(5) Repeat steps 3 and 4 to achieve a change in pitch angle by constantly changing the height of the device 2 and aiming at the center of the light source;
(6) Substitute the corresponding parameters of each group and the position information from PSD into the mathematical model, and calculated the coordinate of the explosion point $\left(X_{c},Y_{c},Z_{c}\right)$.

Then the calculation of Equation (16) leads to Table 3.

**Figure 16:**
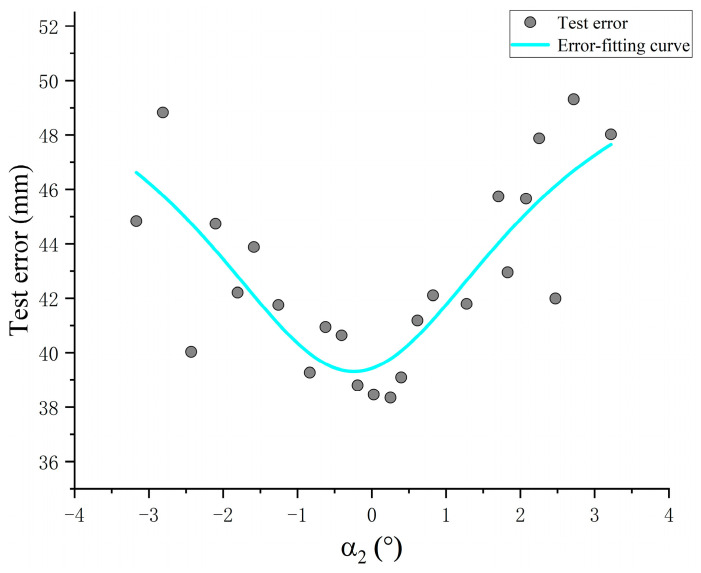
Variation trend of test error on pitch angle.

As shown in the table, the pitch angle $\alpha_{1}$ = −0.303°, $\alpha_{2}$ changes from −3.168° to 3.219°, corresponding to the variation range of the system test error is 38.35 mm −49.31 mm. The minimum test error for pitch angle $\alpha_{2}$ is 0.253°, and maximum test error at $\alpha_{2}$ = 2.719°. The change amplitude of the test error is small. Curve fitting of the data gives Figure 16.

The variation trend of the test error of the system can be clearly observed from the figure. When the pitch angle of one side of the device is varied individually, the closer the angle is to 0°, the smaller the test error of the system is. Since the pitch angles of the two devices change independently and do not affect each other, combined with Figure 7 derived from the simulation, the test error of the system is small within the pitch angle of −2.5°–2.5°, and the average test error is 41.87 mm.

### 4.4. Validation Experiment for the Error Distribution in the Detection Area

**Figure 17:**
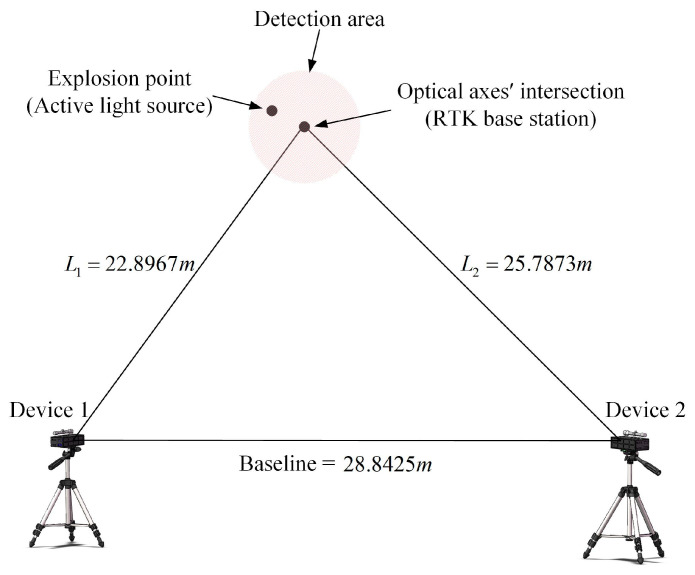
Schematic diagram of the experiment in the detection area.

Setting up the base station pole of the RTK measuring instrument as the optical axes’ intersection, zeroing the pitch angle and roll angle of the device with a spirit level, then measuring and calculating the structural parameters of the system by means of the RTK instrument. The schematic is shown in Figure 17.

As shown in the figure, the system can form a detection area about 4 m × 4 m. The experiment procedure is as follows:

(1) Set up the two devices and the RTK base station and use a level to control the pitch angle and roll angle of the devices to zero, then adjust the two devices so that they are always aimed at the same point on the pole of the RTK base station to achieve optical axis intersection;
(2) Use RTK to measure the length of $L_{1}$ and $L_{2}$ and the baseline, then calculate the azimuth angle, keeping the position of the device constant to keep the system azimuth angle constant;
(3) Place a light source anywhere in the detection area, measure the position of the light source by RTK, and record it as the actual coordinates of the simulated explosion point $\left(X_{w},Y_{w},Z_{w}\right)$;
(4) Use the flash trigger to trigger the light source, use the PSD to detect the position information of the flash, and download the waveform file to the host computer through the acquisition module;
(5) Repeat steps 3 and 4 to simulate the explosion point falling in different positions within the detection area by moving the position of the light source several times;
(6) Substitute the parameters and the position information from the PSD of each group into the mathematical model and calculate the coordinate of the explosion point $\left(X_{c},Y_{c},Z_{c}\right)$.

The test error of the system for each group of explosion points is obtained through Equation (16), as shown in Table 4.

**Figure 18:**
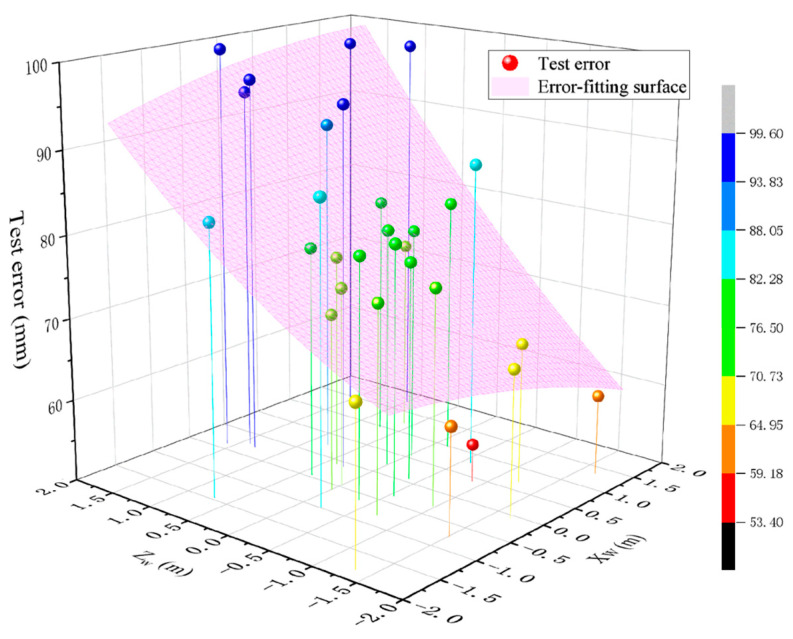
Distribution of the test error in the detection area.

According to the data in the table, with a distance of 25 m, the test error of the system for the coordinates of the explosion points at different positions in the 4 m × 4 m detection area can be obtained. The maximum test error is 99.59 mm, corresponding to the actual coordinate $\left(X_{w},{\;Y}_{w},\;Z_{w}\right)$ = (−0.3776, 0.0955, 1.5875), and the minimum test error is 54.66 mm, corresponding to the actual coordinate $\left(X_{w},Y_{w},Z_{w}\right)$ = (0.4122, 0.0261, −0.8835). A surface fitting of the data yields Figure 18.

In the figure, the fitted surface can be more intuitively observed to obtain the distribution and variation trend of the test error in the detection area. Along the $Z_{w}$-axis from positive to negative, the test error of the system gradually decreases. In the region of the negative half-axis of the *Z*-axis, the closer to the edge of the region, the lower the test error. The change amplitude of the test error in the direction of the $X_{w}$-axis is small. Combined with the error distribution derived from the simulation in Figure 9, it can be verified that, in the detection area, the test accuracy of the system is higher when the explosion points fall in the region of the negative half-axis of the *Z*-axis of the world coordinate system, where the test error in this region is not more than 87.17 mm, with an average of 73.38 mm.

In summary, the results for the analysis and optimization of the structural parameters of the system are presented in Table 5.

## 5. Conclusions

In this paper, for the long-distance spatial positioning technology of the position of the explosion point, a spatial coordinate testing method for explosion points based on a two-dimensional PSD (position-sensitive detector) intersection is proposed. Firstly, the mathematical model of a spatial coordinate testing system based on a PSD intersection is constructed, and the error propagation model is established. Then, the influence of the optical axes’ intersection position as well as the azimuth angle and pitch angle on the test accuracy of the system is simulated and analyzed, and the distribution and variation trend of the error propagation coefficient of the system is obtained. Finally, the test error of the system is obtained through the design experiments for validation. The results show that the system test accuracy is high when the azimuth angle is 20°–50°, the overall error propagation coefficient does not exceed 48.80, and the average test error is 56.17 mm. When the pitch angle is −2.5°–2.5°, the system has a higher test accuracy, with the overall error propagation coefficient not exceeding 44.82, and the average test error is 41.87 mm. The test accuracy of the system is higher when the position of the optical axes’ intersection is chosen to make sure that explosion points fall in the region of the negative half-axis of the $Z_{w}$-axis of the world coordinate system, with an overall error propagation coefficient of less than 44.78 and an average test error of 73.38 mm. Therefore, a reasonable selection of system structure parameters can significantly improve the system test accuracy and optimize the system deployment mode under long-distance field conditions so as to improve the deployment efficiency.

## Figures and Tables

**Table 1:** Instrument information.

Instrumentation	Provider	Type No.	Main Parameters	
PSD	HAMAMATSU, Shizuoka, Japan	C10443-03	Spectral response range	320–1060 nm
			Photosensitivity	−60 mV/μW
			Position detection error	±150 μm
			Position resolution	1.4 μm
Acquisition	TDEC, Chengdu, China	iSD-404 W	Sampling frequency	Max: 2 MHz
RTK	HI-TARGET, Guangzhou, China	V200	Positioning accuracy	Plane: ±8 mm
				Altitude: ±15 mm
Lens	Nikon, Kanagawa, Japan	AF 50 mm f/1.8 D	Focal length	50 mm
			Distortion rate	0.438%

**Table 2:** Test error for the effect of azimuth angle on the system.

	Device 1	Device 2	Test Error
	$\theta_{1}$ (°)	$\theta_{2}$ (°)	E (mm)
1	10.5752	10.5638	57.82
2	27.551	20.3861	56.54
3	14.8183	25.9639	57.58
4	38.4242	26.4696	52.65
5	46.9864	22.2296	55.02
6	77.1736	29.0752	61.08
7	82.0404	22.7545	59.42
8	70.1285	40.7945	61.22
9	73.008	45.7415	87.31
10	77.9635	51.8593	123.73
11	70.0748	70.9365	229.48
12	71.2491	62.4419	152.03
13	65.5684	82.7093	66.74
14	85.9585	72.8724	415.12
15	48.7588	65.5269	83.70
16	42.0829	73.8213	83.32
17	33.4197	77.0674	86.91
18	25.571	68.0733	63.67
19	22.6404	78.834	69.89
20	17.6403	67.2202	63.08
21	22.8052	40.2695	61.93
22	8.5798	48.2914	59.21
23	44.9984	45.3498	58.51
24	34.316	45.4239	50.17
25	45.23	54.2921	63.97
26	45.8272	36.5607	64.04
27	53.0134	47.4595	68.36
28	53.841	55.338	77.98
29	36.318	54.7984	62.61
30	48.1295	34.3691	50.52

**Table 3:** Test error for the effect of pitch angle on the system.

	Device 1	Device 2	Test Error
	$\alpha_{1}$ (°)	$\alpha_{2}$ (°)	E (mm)
1	−0.303	3.219	48.02
2		2.719	49.31
3		2.473	41.99
4		2.253	47.87
5		2.077	45.66
6		1.829	42.96
7		1.706	45.74
8		1.277	41.79
9		0.825	42.11
10		0.615	41.18
11		0.396	39.09
12		0.253	38.35
13		0.026	38.46
14		−0.19	38.80
15		−0.405	40.64
16		−0.622	40.94
17		−0.834	39.26
18		−1.256	41.75
19		−1.586	43.88
20		−1.806	42.21
21		−2.103	44.74
22		−2.43	40.03
23		−2.812	48.83
24		−3.168	44.83

**Table 4:** Test error of the coordinate of the explosion point in the detection area.

	Actual Coordinates			System Testing Coordinates			Test Error
	$X_{w}$ (m)	$Y_{w}$ (m)	$Z_{w}$ (m)	$X_{c}$ (m)	$Y_{c}$ (m)	$Z_{c}$ (m)	E (mm)
1	0.4122	0.0261	−0.8835	0.3734	0.0292	−0.922	54.66
2	−0.3057	0.0594	−0.9778	−0.3725	0.0502	−1.0144	76.17
3	−0.3521	0.0812	−0.5492	−0.4154	0.0446	−0.5988	80.42
4	0.0689	0.0895	−0.3879	0.0141	0.0387	−0.4417	76.80
5	0.9894	0.0601	−0.1365	0.9423	0.0225	−0.2025	81.08
6	−0.6032	0.0968	−0.0027	−0.6595	0.0612	−0.0467	71.45
7	0.2444	0.0995	0.7931	0.1837	0.0486	0.7258	90.63
8	−0.2382	0.1174	1.4077	−0.3191	0.0653	1.3589	94.48
9	0.9704	0.2196	0.7209	0.9211	0.0276	0.7833	79.53
10	−0.2784	0.1054	1.3009	−0.3649	0.0613	1.2588	96.20
11	−0.0881	0.1362	0.3828	−0.1567	0.0583	0.35	76.04
12	−0.4580	0.0902	0.0032	−0.5176	0.0634	−0.041	74.20
13	0.5562	0.0906	−0.0315	0.4919	0.0438	−0.0766	78.54
14	−0.7899	0.2216	−0.7133	−0.8565	0.0658	−0.6785	75.14
15	0.6855	0.1832	−1.2314	0.6347	0.0247	−1.1874	67.21
16	−0.0681	0.0889	−1.6852	−0.1297	0.0427	−1.7136	67.83
17	−0.7443	0.0699	−1.5101	−0.7227	0.0552	−1.451	62.92
18	1.2282	0.0820	0.6127	1.2726	0.0276	0.554	73.60
19	−0.3776	0.0955	1.5875	−0.452	0.0646	1.5213	99.59
20	−0.0973	0.2384	0.2896	−0.0448	0.0542	0.211	94.52
21	0.2198	0.1075	0.0094	0.1499	0.0466	−0.0282	79.37
22	−0.6263	0.1119	−0.3614	−0.698	0.0539	−0.3953	79.31
23	−0.9908	0.0902	−0.2139	−1.0521	0.0767	−0.2751	86.62
24	0.7754	0.0760	−0.5872	0.7098	0.0335	−0.6446	87.17
25	−0.4547	0.1291	0.3808	−0.5152	0.0663	0.3314	78.11
26	1.7395	0.2155	0.989	1.6769	0.0395	0.9142	97.54
27	1.2886	0.0489	1.3997	1.362	0.0519	1.4647	98.04
28	1.3411	0.0977	−1.6641	1.3015	0.0229	−1.709	59.87
29	−1.4613	0.1287	0.6915	−1.4109	0.0437	0.6255	83.04
30	−1.8117	0.2981	−1.329	−1.8549	0.0262	−1.2753	68.92

**Table 5:** Results of the analysis and optimization of the structural parameters of the system.

System Structure Parameters	The Azimuth Angle	The Pitch Angle	Position of the Intersection
Error propagation coefficient	≤48.80	≤44.82	≤44.78
Test error	56.17 mm	41.87 mm	73.38 mm
Optimization	Control the azimuth angle within 20°–50°	Control the azimuth angle within −2.5°–2.5°	Make sure that explosion points fall in the region of the negative half-axis of the *Z_w_*-axis of the world coordinate system

## Data Availability

The data are not publicly available due to the confidentiality of the research projects.
